# The influence of early exposure to vitamin D for development of diseases later in life

**DOI:** 10.1186/1471-2458-13-515

**Published:** 2013-05-28

**Authors:** Ramune Jacobsen, Bo Abrahamsen, Marta Bauerek, Claus Holst, Camilla B Jensen, Joachim Knop, Kyle Raymond, Lone B Rasmussen, Maria Stougaard, Thorkild IA Sørensen, Allan A Vaag, Berit L Heitmann

**Affiliations:** 1 Institute of Preventive Medicine, Bispebjerg and Frederiksberg Hospitals - a part of Copenhagen University Hospital, Nordre Fasanvej 57, DK-2000 Frederiksberg, Denmark; 2 Institute of Clinical Research, University of Southern Denmark, Winsløwparken 19, Odense, DK-5000, Denmark; 3 Danish Veterinary and Food Administration, Ministry of Food, Agriculture and Fisheries, Stationsparken 31-33, Glostrup, DK-2600, Denmark; 4 Institute of Clinical Medicine, Orthopedics and Internal Medicine, Rigshospitalet, Blegdamsvej 9, Copenhagen, DK-2100, Denmark; 5 National Institute of Public Health, University of Southern Denmark, Øster Farimagsgade 5 A, DK-2000 Copenhagen, Denmark

**Keywords:** Vitamin D, Food fortification, Prenatal exposure, Prevention, Type 1 diabetes, Obesity, Fractures

## Abstract

**Background:**

Vitamin D deficiency is common among otherwise healthy pregnant women and may have consequences for them as well as the early development and long-term health of their children. However, the importance of maternal vitamin D status on offspring health later in life has not been widely studied. The present study includes an in-depth examination of the influence of exposure to vitamin D early in life for development of fractures of the wrist, arm and clavicle; obesity, and type 1 diabetes (T1D) during child- and adulthood.

**Methods/design:**

The study is based on the fact that in 1961 fortifying margarine with vitamin D became mandatory in Denmark and in 1972 low fat milk fortification was allowed. Apart from determining the influences of exposure prior to conception and during prenatal life, we will examine the importance of vitamin D exposure during specific seasons and trimesters, by comparing disease incidence among individuals born before and after fortification. The Danish National databases assure that there are a sufficient number of individuals to verify any vitamin D effects during different gestation phases. Additionally, a validated method will be used to determine neonatal vitamin D status using stored dried blood spots (DBS) from individuals who developed the aforementioned disease entities as adults and their time and gender-matched controls.

**Discussion:**

The results of the study will contribute to our current understanding of the significance of supplementation with vitamin D. More specifically, they will enable new research in related fields, including interventional research designed to assess supplementation needs for different subgroups of pregnant women. Also, other health outcomes can subsequently be studied to generate multiple health research opportunities involving vitamin D. Finally, the results of the study will justify the debate of Danish health authorities whether to resume vitamin D supplementation policies.

## Background

More and more attention is now being paid to the importance of prenatal nutrition and the long-term influence of the mother’s nutrition for long-term health of the progeny, including the mother’s nutritional status prior to conception. The importance of this approach is supported by the newly released draft report from the Scientific Advisory Committee on Nutrition, commissioned by the British Health Department and the Food Standards Agency in 2010 [1]. The report highlights the need for a better understanding of the influence of nutritional exposure during critical time periods in human development and the type of dietary interventions that might be employed to improve child nutrition and elicit long-term health benefits. The present study addresses these issues in relation to vitamin D.

### Vitamin D

Vitamin D is a fat-soluble vitamin that is necessary for the human body. It plays an important role in a wide variety of chronic health conditions. The discovery of high-affinity vitamin D receptors (VDR) in many organs and cell types has led to an increasing focus on the role of vitamin D as a steroid hormone with significant influences on cell metabolism and proliferation. Widely distributed in body tissues, a VDR, once bound to a vitamin D ligand, travels to the nucleus where it binds to sites within the human genome and consequently influences the activity, or expression, of individual genes. A recent study reported identification of as many as 2,776 sites of vitamin D receptor binding and 229 genes whose expression was directly linked to changes in vitamin D levels [2]. Many of these genes are involved in bone and muscle development, metabolism and immune function. Consequently, low vitamin D influences bone health, body growth and composition, and also impacts on immunity [3,4]. Furthermore, as proposed in 2001, a critical window may exist during which vitamin D levels have a persisting impact on health outcomes [5]. Low vitamin D during gestation and in early life may therefore play an important role for susceptibility to bone fragility, obesity and autoimmune diseases later in life [6].

Vitamin D is synthesized in the skin when exposed to ultraviolet-B radiation from the sun. 7-dehydro-cholesterol is converted to cholecalciferol (vitamin D3) which is then, in kidneys, modified further to form the active vitamin binding to VDR. Animal tissues consumed as food provide additional sources of this hormone-like vitamin, while plant foods may provide the less potent form of vitamin D: ergocalciferol (vitamin D2) [7]. Although adequate exposure to UVB sunlight is paramount to an ample supply of vitamin D, oral intake, augmented by both fortification and supplementation, is necessary to maintain baseline stores in populations at high latitudes were the sun can only drive dermal synthesis of vitamin D from April to October [8]. It has been shown that fortification is particularly relevant for those with low stores, e.g. pregnant women [9].

### Importance of fetal vitamin D on health later in life

Vitamin D insufficiency is highly prevalent in both developed and developing societies. It is estimated that one in seven, or about 860 million people around the world, have vitamin D deficiency or insufficiency [9]. With the high general prevalence of vitamin D deficiency, the status of pregnant and lactating women is of particular concern. Low levels may have consequences for not only the mother but also for the early development and long-term health of her offspring, since fetal stores of vitamin D depend on maternal concentrations [10]. Pregnancy is a time of particular susceptibility to vitamin D deficiency, as both maternal and fetal demands for the vitamin need to be met [11,12]. The development of tissues and organs at different stages of fetal growth makes timing of deficiency during gestation of particular importance, as the interaction between differential development and deficiency can determine the effect on the child. For instance, mothers with babies born between February and April will have had low sun-related UVB exposure, and hence, lower vitamin D exposure for their offspring during the third trimester, while babies born between May and July will have had low exposure in their second trimester and those born between August and October will have had low exposure during the first trimester. Adverse health effects of low vitamin D may therefore be most evident in organs or tissues whose development occurs in these trimesters, e.g. obesity may be particularly associated with low vitamin D during the third trimester when adipose tissue is formed; fractures may be particularly associated with low vitamin D during the third and/or second trimester [13]; and T1D with low vitamin D during the first trimester.

To date, the relationship between vitamin D status during fetal life and long-term health has not been widely examined, and interventions are generally lacking. A few studies suggest that low fetal vitamin D may reduce bone mineral content, consequently leading to fragility fractures [14]. Possibly it also affects metabolic functioning and consequently leads to obesity [15]. Some studies have found that adult obesity varies as a function of month of birth and that subjects born from winter to spring become more obese, possibly due to low vitamin D levels from restricted maternal sun exposure during the third trimester of pregnancy [16]. Furthermore, a recent study reported that a low level of maternal vitamin D during pregnancy was related to high fat and low muscle in offspring at ages 5–9 [15]. Although mechanisms relating low perinatal vitamin D to later obesity are not well known, proposed mediators include epigenetic changes leading to initiation of adipogenesis, or induction of permanent functional changes in hypothalamic appetite regulation [17,18]. Furthermore, there are studies showing that low fetal vitamin D may lead to persistent changes in the immune system, thus possibly influencing the risk of developing autoimmune diseases such as T1D [14,19,20].

Most evidence linking prenatal nutrition to later disease comes from animal studies, or from natural experiments, such as the Dutch Famine during World War II or the Great Chinese Famine during the Great Leap Forward. Both studies suggest that prenatal malnutrition strongly impacts adult health, although neither indicates specific effects from the lack of vitamin D. The effect of vitamin D levels during gestation, thus, has never previously been followed in large populations. Furthermore, large-scale interventional studies are needed to examine how vitamin D exposure during pregnancy affects long-term health outcomes of offspring. Such studies may never be conducted due to the associated high financial and logistical costs. To study the influence of seasonal variation and timing of fetal exposure in relation to later effects of vitamin D on health outcomes, one requires even larger numbers of subjects to be followed over long time.

### Objectives and hypothesis

The present study relies on a unique societal experiment in which all margarine and low fat milk products consumed by the Danish population were fortified with vitamin D, during two distinct time periods: in 1961–1985 fortifying margarine with vitamin D was mandatory in Denmark and in 1972–1976 low fat milk fortification was allowed. The present study also relies on the fact that in Danish national administrative and large research databases data can be linked at the individual level. This allows for the examination of the effects of exposure to additional vitamin D via fortified foods during fetal life in relation to later development of particular diseases. Initially the present study will include an in-depth examination of the influence of exposure to vitamin D during pregnancy for development of fractures of the wrist, forearm, upper arm and shoulder, as well as fractures of ankle; development of obesity; and development of T1D during child- and adulthood. Furthermore, the large number of subjects that will be followed will ensure that the effects of vitamin D exposure in relation to the timing of the seasons of gestational development also can be examined. Additionally, a validated method will be used to determine neonatal vitamin D status using stored dried blood spots (DBS) from individuals who develop the aforementioned disease entities as adults and their time and gender-matched controls. The main hypothesis is that low levels of vitamin D during critical phases of development have adverse effects on the long term risk of developing the above mentioned disease outcomes, and that exposure to extra vitamin D from food fortification has beneficial effects preventing the mentioned diseases.

## Methods/design

### Study populations

**Figure 1:**
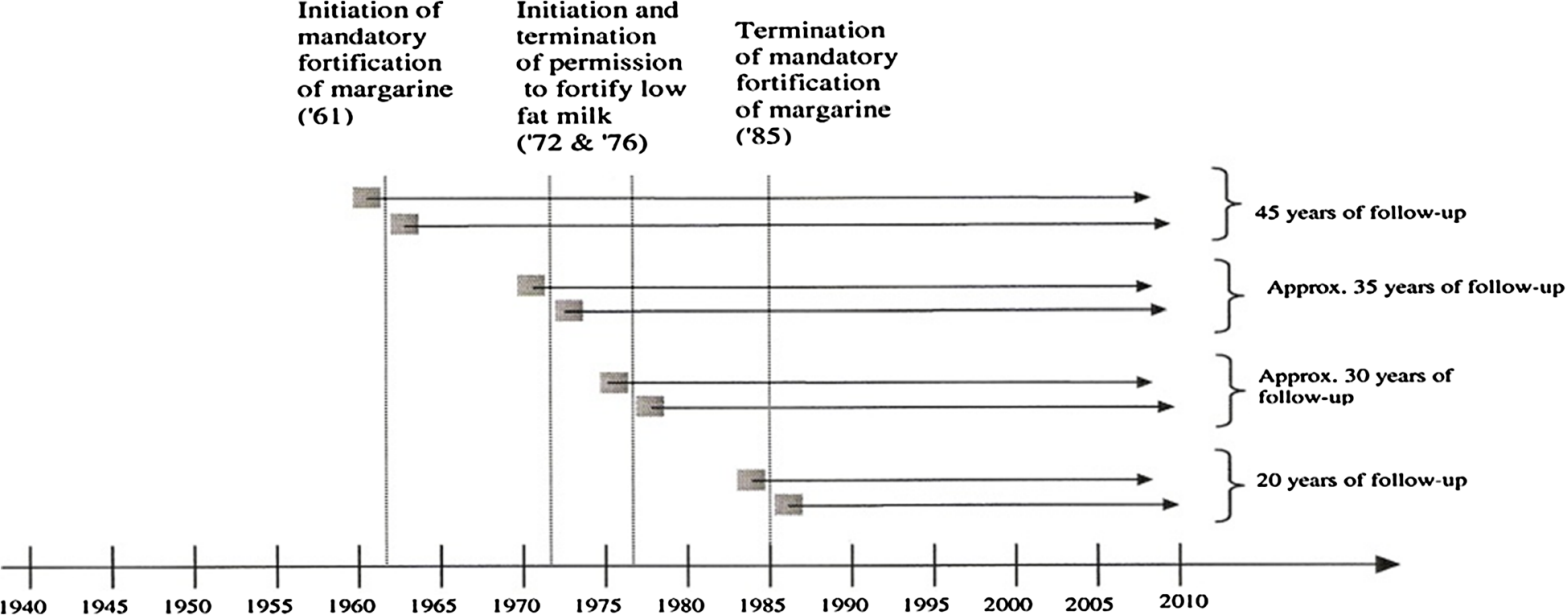
**Study cohorts. **The figure provides examples of designs for examining the effects of vitamin D fortification on development of diseases for a follow-up period of up to 50ὕyears. The birth (grey boxes with allowance for short “wash-out period” in between) and follow-up years (arrows) of individuals from subgroups exposed before and after birth are given in relation to initiation or termination of vitamin D fortification.

In Denmark, from 1961 to 1985 vitamin D fortification of margarine was mandatory (1.25ὕμg/100ὕg), and from 1972 to 1976 fortification of low fat milk (2.5-3.8ὕμg/100ὕgὕmilk) was permitted. The vitamin D fortification of low fat milk was not mandatory and therefore not all low fat milk distributed in Denmark in the time period was fortified with vitamin D. Unfortunately, there are no national statistics indicating the proportion of milk consumed that was fortified with vitamin D. Nevertheless, the two well-defined time periods of vitamin D fortification provide a natural framework for comparing the effects of early exposure to vitamin D on the development of diseases later in life amongst exposed and non-exposed adjacent birth cohorts (Figure 1).

The study relies upon the well-defined adjacent time windows, as well as the complete registration of every citizen in Denmark via the Danish Civil registration System. Every citizen in Denmark alive April 1^st^ 1968 is assigned a civil registration (CPR) number, and this number can be used to link information, on an individual level, in the Danish birth, patient and medical registries; social and ethnic registries; and clinical and other large databases. Consequently, the study will include approx. 3 mio. individuals from nationwide birth cohorts between the 1930ies and today.

### Databases

In a first effort, we will examine influence of additional early exposure to vitamin D on subsequent development of fractures, obesity, and T1D.

#### Fractures

Using the CPR numbers, individuals will be individually linked to the Danish National Patient Registry for incident and recurrent diseases. The register contains information about hospital contacts, including diagnosis codes and procedure codes for all treatment received at Danish hospitals [21]; after 1994, outpatient treatment is also captured by the register. From 1977–1993, diagnoses were classified according to the WHO International Classification of Diseases (ICD-8) and from 1994 onwards according to ICD-10. The main study outcomes are fractures at wrist and hand level (ICD-8: 814–817; ICD-10: S62); fractures of forearm (ICD-8: 813; ICD-10: S52); fractures of shoulder and upper arm (ICD-8: 810–812; ICD-10: S42); and fracture of ankle (ICD-8: 824; ICD-10: S82.5, S82.6. S82.8).

#### Childhood overweight and obesity

The Copenhagen School Health Record Registry (CSHRR) includes every student attending a primary school in Copenhagen Municipality since birth in year 1930 through 1990 and comprises more than 370,000 records, which have been computerized for research purposes [22-24]. Overweight and obesity will be calculated from weight and height, which were measured at age 6–7 and at age 13–14 throughout the time period, but also annually until 1984. The CSHRR comprises approx. 10% of all Danes from each birth cohort. Birth weight, birth complications, gestational diabetes, pre-pregnancy weight and gestational and maternal age are retrievable from the National Medical Birth Registry. Data are available from 1973 onward.

#### T1D

The Danish Childhood Diabetes Registry covers all children age 0–15 at diagnosis with T1D. The registry covers all patients born since 1981 [25].

Currently a second phase of the project is planned. Finances are being applied for to also include outcomes such as type 2 diabetes (T2D), early cancer of the breast and colon, pre-eclampsia, dental diagnosis such a carries, psychiatric disorders such as schizophrenia, paranoid psychoses, mood disorders, organic mental disorders, and abuse of alcohol and other psychoactive substances. Information on development of these outcomes can be retrieved from the National Diabetes Registry, National Patient Registry, and the Danish Psychiatric Research Registry. In the future, a third phase will be planned and will examine association between additional exposure to vitamin D in fetal life and later development of other immunological disorders, including severe asthma, Crohn’s disease, inflammatory bowel diseases, and Juvenile idiopathic arthritis.

#### Vitamin D status (25(OH)D3)

Since May 1, 1981, routinely collected neonatal DBS samples taken by heel prick 48–72 hours after birth have been collected for all newborns in Denmark. After routine screening for congenital disorders, residual DBS cards are stored in the Biological Specimen Bank for Neonatal Screening at Statens Serum Institute [26]. Punches of stored surplus card samples will be used to measure neonatal 25(OH)D3 [27]. The assay detection method is a highly sensitive liquid chromatography tandem mass spectroscopy coupled with multiple reactant monitoring. Previous studies have shown that storage times of more than 20 years do not bias inter-individual variation in concentrations for a given birth cohort [28].

#### Education, unemployment, income and ethnicity

This information will be retrieved from the Statistics Denmark Fertility Database, which includes information on subjects of fertile age born since 1930 who still reside in Denmark. Social information is available for anyone born since 1981.

Data from the National Patient Registry, the CSHRR, The Danish Childhood Diabetes Registry, and the results of the DBS cards analyses will be merged and sent to Statistics Denmark for merging with their databases. Statistics Denmark will then create the relevant sub-datasets to be analyzed on-line.

### Exposure assessment

Four main analyses of exposure related to initiation and termination of vitamin D fortification will be considered for each of the disease outcomes:

1. perinatal exposure to vitamin D for offspring;

2. seasonal variation in vitamin D exposure in different pregnancy trimesters;

3. exposure to vitamin D adjusted for genetic and common environmental influences;

4. levels of vitamin D (25(OH)D3) measured in infant blood from DBS cards.

#### Perinatal exposure

Development of the disease outcomes will be compared for children born around the time when mandatory vitamin D fortification was initiated and terminated. The hypothesis is that individuals born 1–2 years after commencement of mandatory fortification (1961) and thus exposed to extra vitamin D during fetal development and for the next 24–25 years until 1985 (where fortification was ended), although unexposed thereafter, will have a lower lifetime risk of developing the aforementioned disease entities compared to individuals born 1 –2 years before fortification. Although also exposed to fortification for 24 – 25 years and unexposed thereafter, the latter group was unexposed during fetal life. Likewise, individuals born 1–2 years after mandatory fortification terminated in 1985, and who were unexposed to vitamin D both during fetal development and subsequently, will have a higher risk of later developing the diseases compared to those exposed during fetal life. Similar comparisons of disease development can be made in relation to higher exposure to vitamin D from 1972–1976, when fortified low fat milk was permitted.

#### Seasonal variation in vitamin D exposure

The extra vitamin D from fortification is expected to be of particular relevance when stores are low [9]. To study seasonal variations, large datasets are needed and will be created by including individuals from 2–4 birth cohorts born before and after fortification. Whether or not exposure to vitamin D fortification in different gestational trimesters is important will be examined. For instance, influence of low vitamin D on obesity and adipose tissues may be particularly important for children whose third gestation periods occur during winter, while influence of low vitamin D on autoimmune diseases may be particularly important for children whose first gestational trimester occurs during winter.

#### Common environment and genetics adjusted exposure

To supplement the above mentioned cohort analyses, a nested sibling pair analysis, with one sibling identified before fortification was initiated (or terminated) and a gender-matched sibling identified after, will be applied for each of the analyses specified above. This design takes into account the common environment and genetics by matching on maternal genes and (assuming no change of partner) paternal genes. The genotypes of the two siblings may still differ, but on average with considerably less differences than between two unrelated subjects. This design assumes lack of birth order effects, but this can be partially tested by comparing results in sib-pairs in which the 1^st^ or 2^nd^ is the exposed dependent on whether they were born before, during or after the fortification period. The comparability of early life experiences in each sibling pair may reduce unmeasured confounding.

#### 25(OH)D3 level from DBS cards

Because the 25(OH)D3 levels in the newborn blood reflects the vitamin D status of the newborn as well as the mother at the end of pregnancy [29], measurement of 25(OH)D3 levels in DBS will serve as validation for the effect of fortification for incident cases. A control, matched for gender, exact date of birth, gestational age, being alive and with none of the aforementioned diseases will be selected and 25(OH)D3 measured. Date of birth matching criterion means that degradation of 25(OH)D3 is unlikely to bias results.

### Power calculation

In relation to long-term health effects of initiation (or termination) of the fortification, analyses including continuous outcomes, such as BMI, will not present power problems. For incidence of obesity, fractures and T1D, calculating the least detectable excess risk is relevant. The power calculation is done generically in a worst-case scenario using a dichotomous outcome with a low prevalence. The least detectable excess risk is calculated assuming that either one (at least 50,000 individuals) or two birth cohorts are included. As the follow-up period varies, the final prevalence (= incidence × duration) will be used and varied from 1-8%. The significance level is α = 0.05 and power β = 0.80. Table 1 shows the least detectable relative risk of outcome after change in fortification.

**Table 1:** Least detectable relative risk of outcomes in relation to exposure to foods fortified with vitamin D

**N (exposed/non-exposed)**	**Prevalence of outcome (fractures, T1D, obesity)**				
	**0.5%**	**1%**	**2%**	**4%**	**8%**
50,000/50,000	1.27	1.19	1.13	1.09	1.07
100,000/100,000	1.19	1.13	1.09	1.07	1.05

For analyses using DBSs, power calculations were performed under 3 scenarios related to the fraction of the exposure variance explained including covariates: no adjustment, 30% explained or 60% explained. Table 2 gives the least detectable hazard ratios (for fractures and diabetes) or odds ratios (for BMI) of outcome related to 1 SD difference in 25(OH)D3 for a sample size of 1000 cases and 1000 controls using α?=?0.05 and β?=?0.80.

**Table 2:** Least detectable hazard ratios (for fractures and diabetes) or odds ratios (for BMI) of outcome related to 1 SD difference in 25(OH)D3 for a sample size of 1000 cases and 1000 controls

**Least detectable Hazard (HR) or Odds rate (OR) ratios related to 1 SD difference in 25(OH)D3**	**1000 cases (obesity, diabetes or fractures) and 1000 time- and gender-matched controls**		
	**No covariates**	**30% variance explained**	**60% variance explained**
OR ratio	1.13	1.16	1.22
HR ratio	1.09	1.11	1.15

### Statistical analyses

For the time trend analyses (i.e. to compare time to debut of a disease) of fractures and T1D, cohort analyses with all individuals born in the respective years entering the cohorts at time of birth, and the key covariates being the year/month of birth, will be conducted using Cox regression models. For the time trend analyses of obesity, similar cohort analyses will be conducted, where linear regression models will be used for the BMI as a continuous trait and logistic regression models will be used for the categorical variables (i.e. overweight and obesity), again with the year/month of birth as the key covariate. Secular trends within multiple birth cohorts used before and after change in fortification will be addressed using appropriate adjustments. Gender differences will also be considered.

Case–control analyses are envisaged for a comparison of blood spot vitamin D levels between subjects with a disease outcome and subjects who have not developed this outcome. These analyses still are based on a cohort - the entire population who has a blood spot card available, but the sampling design will be a case-cohort design, where information about the exposure will be collected only for cases developed after entry in the cohort and matched controls, randomly selected within specified strata. These data can be analysed using the Cox regression models for the event-type outcome and the logistic regression models for the dichotomous outcomes. The adjustment for possible confounders will be of particular importance in these analyses.

### Legal and ethical aspects

All relevant databases are accessible and the collection of data in these registries has been undertaken in accordance with the generally accepted ethical principles for informed consent and according to the Declaration of Helsinki. Furthermore, all data collection has been approved by the relevant ethical committees in accordance with Danish law. The access and linkage permission from the Danish Data Protection Agency to provide access to the Danish Civil Registration System, where individual civil registration numbers are stored, has already been granted (J. no.: 2010-41-4485). This permission includes merging civil registration numbers with different nationwide disease registers. Statistics Denmark will create the relevant sub-datasets, but before access is allowed the civil registration numbers are hidden. Permission to access and analyze the DBS samples from the Biological Specimen Bank for Neonatal Screening has also been granted by the Ethical Committee D of the Capital Region of Denmark (J. no.: H-3-2011-126).

## Discussion

### Strengths and limitations

The study takes advantage of a vitamin D fortification policy implemented in Denmark almost fifty years ago, and nests this “societal experiment” in a setting where the effect of conditions from conception (and before) for development of certain diseases can be followed among individuals from birth through adulthood. The study is also possible due to complete registration of every citizen in Denmark via a civil registration number. This number can be linked, on an individual level, to the Danish birth, patient and medical registries; social and ethnic registries; and clinical and other large databases. The comprehensive linkage of different datasets makes it possible to study the mediation, confounding or modification of effects of vitamin D for selected conditions through the development of sophisticated models in which individuals can be followed for periods of up to 50 years. Moreover, the large number of subjects that will be followed ensures that the effects of vitamin D exposure in relation to the timing of the seasons and gestational development can also be examined. Nevertheless, the proposed study design implies several uncertainties, including so-called ecological fallacies, addressed below.

Fortification supplied 15% of vitamin D intake on average, which may be considered low, particularly when the sun exposure is not ample. However, vitamin D fortification is especially beneficial when vitamin D status is low [9], e.g. when the previous 6 months of UVB radiation from sun exposure in Denmark was too weak to induce vitamin D conversion in the skin [30]. The use of 50,000-100,000 individuals from adjacent birth years in the general population studies of events like fractures and diagnosis of T1D, a pivotal strength of this study, makes it possible to detect even minor effects of vitamin D.

Food disappearance statistics show that margarine intake decreased and low fat milk slightly increased during the period covered. Nevertheless, changes of this nature are not expected to influence results, because the intervention group and the control group will have been born in adjacent years. Moreover, any societal changes succeeding the initiation of mandatory fortification in 1961 or its termination in 1985 may influence the intervention and control groups similarly. Thus, we find it unlikely that changes in occurrence in the outcomes associated with the introduction and cessation of fortification will lead to spurious conclusions.

We do not collect any individual data on vitamin D intake. In contrary, the individuals in the database are unselected in relation to vitamin D exposure as well as to later disease occurrence. While assuming no ecological fallacies, this in fact may be considered a very strong feature of the present study. Neither confounding from external or internal factors, nor the lack of individual data on vitamin D intake, is a prerequisite for the study, as the influences of lifestyle differences (e.g. use of infant formula, individual diet intake, use of supplements) or societal changes (e.g. in breast feeding, fortified infant formulas, sun exposure) are independent of the vitamin D intervention.

We acknowledge, however, that the differences in prenatal exposure by any subsequent age imply a more simple difference in duration of exposure. Therefore, we will conduct robustness analyses where the age scale is adjusted so that the duration of exposure becomes the same and the difference then only whether the exposure was commenced/ended prenatal or postnatal.

Moreover, there might be regional and urban/rural differences in vitamin D intake. As information on individual’s place of residence is available from Statistics Denmark, it will be included into analyses.

As diet is usually different between families with higher socioeconomic status and those with lower status [31], maternal socioeconomic status may have affected margarine/milk intake and thus exposure to vitamin D. Information on socioeconomic position, education and ethnicity is available from Statistics Denmark and can be controlled for. Adjustments will also be made for maternal age, parity, gestational age and birth weight, which is information available from the birth and patient registries and Statistics Denmark (since 1973, 1977 & 1980, respectively).

Finally, the uncertainties related to lack of knowledge about real vitamin D status in mother and offspring may be eliminated by assessing the measurement of infant blood vitamin D levels in those developing the three disease entities and comparing to time and gender-matched controls to examine actual exposure differences at birth.

### Implications

Unlike many other nutritional deficiencies, low vitamin D status is prevalent among industrialized populations, and, according to Danish health authorities, about half of all adults have vitamin D insufficiency (below 50 nmol/l) [32]. Adequate exposure to UVB sunlight is paramount importance for supply of vitamin D, but oral intake, augmented by fortification and supplementation, is necessary to maintain baseline stores, particularly in winter when sunlight is limited [8,33,34]. Thus, as it is clear that vitamin D status of Danes needs to be improved, the Danish health authorities currently debate, whether to resume vitamin D fortification of food [35]. The complete lack of knowledge on the general health effects of fortified foods is an important issue in this regard. Our results will significantly increase current understanding of the importance of early vitamin D intake for long-term health and the occurrence of diseases. The results will also provide solid quantitative justification whether to resume vitamin D fortification. From a public health perspective, the potential to prevent common chronic diseases via low-cost, simple and safe food fortification is an attractive option.

Additionally, we expect that the results of the study will open new research opportunities for other scientists and fields, e.g. human intervention studies to determine the level of vitamin D supplementation necessary to reduce susceptibility to diseases later in life and concomitant basic research exploring the biological mechanisms that may be involved. Because it is possible to obtain information in Denmark on individual health and social parameters via linkage to health registries using a unique personal identification number, many opportunities for collaboration will arise in relation to health aspects, e.g. growth and pubertal development, possibility related to vitamin D exposures in early life, perhaps even before conception, in addition to the outcomes studied. If and when our nationwide model is in operation for the three diseases studied, the approach used and the inherent methodologies applied can easily be adapted to study other health outcomes.

## Conclusion

The present study relies on a unique societal experiment in which vitamin D fortification of all margarine consumed by the Danish population was mandatory during two distinct time periods. It thus allows examination of the effects of low vitamin D during fetal life in relation to later development of particular diseases. Results from this study are likely to considerably increase our current understanding of the significance of early vitamin D intake in relation to later health. Besides implications for research in the field, it may also inform the current debate in Denmark on whether to resume vitamin D food fortification.

## Competing interests

The authors declare that they have no competing interests.

## Authors’ contribution

RJ participated in the data collection and helped to draft the manuscript. BA contributed to the study design and helped to draft the manuscript. MB helped to draft the manuscript. CH was responsible for statistical support and helped to draft the manuscript. CBJ participated in the data collection and helped to draft the manuscript. JK participated in the data collection and helped to draft the manuscript. KR participated in the data collection and helped to draft the manuscript. LBR contributed to the study design and helped to draft the manuscript. MS participated in the data collection and helped to draft the manuscript. TIAS contributed to the study design and helped to draft the manuscript. AV contributed to the study design and helped to draft the manuscript. BLH conceived the study, was responsible for its design and coordination and drafted the manuscript. All authors read and approved the final manuscript.

## Pre-publication history

The pre-publication history for this paper can be accessed here:

http://www.biomedcentral.com/1471-2458/13/515/prepub

## References

[B1] Williams AF Aggett P Anderson AS Reader RF Jackson AA Key T The SACN Subgroup on Maternal and Child Nutrition (SMCN): the influence of maternal, fetal and child nutrition on the development of chronic disease in later life Scientific Advisory Committee on Nutrition report http://www.sacn.gov.uk/pdfs/sacn_early_nutrition_final_report_20_6_11.pdf . Accessed 20-11-2012

[B2] Ramagopalan SV Heger A Berlanga AJ Maugeri NJ Lincoln MR Burrell A A ChIP-seq defined genome-wide map of vitamin D receptor binding: associations with disease and evolution Genome Res 2010 20 1352 1360 20736230 10.1101/gr.107920.110 PMC2945184

[B3] Hamilton B Vitamin D and human skeletal muscle Scand J Med Sci Sports 2010 20 182 190 19807897 10.1111/j.1600-0838.2009.01016.x PMC2860762

[B4] von Hurst PR Stonehouse W Coad J Vitamin D supplementation reduces insulin resistance in South Asian women living in New Zealand who are insulin resistant and vitamin D deficient - a randomised, placebo-controlled trial Br J Nutr 2010 103 549 555 19781131 10.1017/S0007114509992017

[B5] McGrath J Does ‘imprinting’ with low prenatal vitamin D contribute to the risk of various adult disorders? Med Hypotheses 2001 56 367 371 11359362 10.1054/mehy.2000.1226

[B6] Hypponen E Boucher BJ Avoidance of vitamin D deficiency in pregnancy in the United Kingdom: the case for a unified approach in National policy Br J Nutr 2010 104 309 314 20594390 10.1017/S0007114510002436

[B7] Lips P Vitamin D physiology Prog Biophys Mol Biol 2006 92 4 8 16563471 10.1016/j.pbiomolbio.2006.02.016

[B8] Brot C Vestergaard P Kolthoff N Gram J Hermann AP Sorensen OH Vitamin D status and its adequacy in healthy Danish perimenopausal women: relationships to dietary intake, sun exposure and serum parathyroid hormone Br J Nutr 2001 86 Suppl 1 S97 S103 11520426 10.1079/bjn2001345

[B9] Hypponen E Power C Hypovitaminosis D in British adults at age 45 y: nationwide cohort study of dietary and lifestyle predictors Am J Clin Nutr 2007 85 860 868 17344510 10.1093/ajcn/85.3.860

[B10] Hollis BW Wagner CL Nutritional vitamin D status during pregnancy: reasons for concern CMAJ 2006 174 1287 1290 16636329 10.1503/cmaj.060149 PMC1435950

[B11] Hollis BW Pittard WB III Evaluation of the total fetomaternal vitamin D relationships at term: evidence for racial differences J Clin Endocrinol Metab 1984 59 652 657 6090493 10.1210/jcem-59-4-652

[B12] Lewis S Lucas RM Halliday J Ponsonby AL Vitamin D deficiency and pregnancy: from preconception to birth Mol Nutr Food Res 2010 54 1092 1102 20440696 10.1002/mnfr.201000044

[B13] Abrahamsen B Heitmann BL Eiken PA Season of birth and the risk of hip fracture in danish men and women aged 65+ Front Endocrinol (Lausanne) 2012 3 2 22645516 10.3389/fendo.2012.00002 PMC3355842

[B14] Javaid MK Crozier SR Harvey NC Gale CR Dennison EM Boucher BJ Maternal vitamin D status during pregnancy and childhood bone mass at age 9 years: a longitudinal study Lancet 2006 367 36 43 16399151 10.1016/S0140-6736(06)67922-1

[B15] Krishnaveni GV Veena SR Winder NR Hill JC Noonan K Boucher BJ Maternal vitamin D status during pregnancy and body composition and cardiovascular risk markers in Indian children: the mysore Parthenon study Am J Clin Nutr 2011 93 628 635 21228264 10.3945/ajcn.110.003921 PMC3407368

[B16] Levitan RD Masellis M Lam RW Kaplan AS Davis C Tharmalingam S A birth-season/DRD4 gene interaction predicts weight gain and obesity in women with seasonal affective disorder: a seasonal thrifty phenotype hypothesis Neuropsychopharmacology 2006 31 2498 2503 16760922 10.1038/sj.npp.1301121

[B17] Foss YJ Vitamin D deficiency is the cause of common obesity Med Hypotheses 2009 72 314 321 19054627 10.1016/j.mehy.2008.10.005

[B18] Kong J Li YC Molecular mechanism of 1,25-dihydroxyvitamin D3 inhibition of adipogenesis in 3T3-L1 cells Am J Physiol Endocrinol Metab 2006 290 E916 E924 16368784 10.1152/ajpendo.00410.2005

[B19] Harvey L Burne TH McGrath JJ Eyles DW Developmental vitamin D3 deficiency induces alterations in immune organ morphology and function in adult offspring J Steroid Biochem Mol Biol 2010 121 239 242 20304054 10.1016/j.jsbmb.2010.03.050

[B20] Hypponen E Laara E Reunanen A Jarvelin MR Virtanen SM Intake of vitamin D and risk of type 1 diabetes: a birth-cohort study Lancet 2001 358 1500 1503 11705562 10.1016/S0140-6736(01)06580-1

[B21] Nickelsen TN Data validity and coverage in the Danish national health registry. a literature review Ugeskr Laeger 2001 164 33 37 11810794

[B22] Baker JL Olsen LW Andersen I Pearson S Hansen B Sorensen TI Cohort profile: the Copenhagen school health records register Int J Epidemiol 2009 38 656 662 18719090 10.1093/ije/dyn164 PMC2722813

[B23] Baker JL Sorensen TI Obesity research based on the Copenhagen school health records register Scand J Public Health 2011 39 196 200 21775383 10.1177/1403494811399955

[B24] Bua J Olsen LW Sorensen TI Secular trends in childhood obesity in Denmark during 50 years in relation to economic growth Obesity (Silver Spring) 2007 15 977 985 17426333 10.1038/oby.2007.603

[B25] Carstensen B Kristensen JK Ottosen P Borch-Johnsen K The Danish national diabetes register: trends in incidence, prevalence and mortality Diabetologia 2008 51 2187 2196 18815769 10.1007/s00125-008-1156-z

[B26] Norgaard-Pedersen B Hougaard DM Storage policies and use of the Danish newborn screening biobank J Inherit Metab Dis 2007 30 530 536 17632694 10.1007/s10545-007-0631-x

[B27] Eyles DW Morley R Anderson C Ko P Burne T Permezel M The utility of neonatal dried blood spots for the assessment of neonatal vitamin D status Paediatr Perinat Epidemiol 2010 24 303 308 20415760 10.1111/j.1365-3016.2010.01105.x

[B28] Eyles D Anderson C Ko P Jones A Thomas A Burne T A sensitive LC/MS/MS assay of 25OH vitamin D3 and 25OH vitamin D2 in dried blood spots Clin Chim Acta 2009 403 145 151 19232332 10.1016/j.cca.2009.02.005

[B29] Eising S Svensson J Skogstrand K Nilsson A Lynch K Andersen PS Type 1 diabetes risk analysis on dried blood spot samples from population-based newborns: design and feasibility of an unselected case–control study Paediatr Perinat Epidemiol 2007 21 507 517 17937736 10.1111/j.1365-3016.2007.00846.x

[B30] Barger-Lux MJ Heaney RP Effects of above average summer sun exposure on serum 25-hydroxyvitamin D and calcium absorption J Clin Endocrinol Metab 2002 87 4952 4956 12414856 10.1210/jc.2002-020636

[B31] Laitinen S Rasanen L Viikari J Akerblom HK Diet of Finnish children in relation to the family’s socio-economic status Scand J Soc Med 1995 23 88 94 7676224 10.1177/140349489502300203

[B32] Brot C Darsø P Forebyggelse, diagnostik og behandling af D-vitaminmangel - Baggrundsnotat [Prevention, diagnostics and treatment of vitamin D deficiency - Background information] http://www.regionh.dk/NR/rdonlyres/A8BB90B7-355C-49C7-961A-312411A02861/0/Dvitaminbagrundsnotat.pdf Accessed 20-11-2012

[B33] Glerup H Mikkelsen K Poulsen L Hass E Overbeck S Thomsen J Commonly recommended daily intake of vitamin D is not sufficient if sunlight exposure is limited J Intern Med 2000 247 260 268 10692090 10.1046/j.1365-2796.2000.00595.x

[B34] McKenna MJ Freaney R Byrne P McBrinn Y Murray B Kelly M Safety and efficacy of increasing wintertime vitamin D and calcium intake by milk fortification QJM 1995 88 895 898 8593549

[B35] Mosekilde L Brot C Hyldstrup L Mortensen LS Molgard C Rasmussen SE The vitamin D status of the Danish population needs to be improved Ugeskr Laeger 2005 167 895 897 15789843

